# Belowground seed and bud banks play complementary roles in the potential recruitment of dominant macrophyte communities in a Yangtze River-connected floodplain wetland

**DOI:** 10.3389/fpls.2022.1075496

**Published:** 2022-12-06

**Authors:** Xin-Sheng Chen, Ying Huang, Yun-He Cai, Zhi-Yong Hou, Zheng-Miao Deng, Feng Li, Ye-Ai Zou, Yong-Hong Xie

**Affiliations:** ^1^ Dongting Lake Station for Wetland Ecosystem Research, Institute of Subtropical Agriculture, The Chinese Academy of Sciences, Changsha, China; ^2^ School of Resources and Environmental Engineering, Anhui University, Hefei, China; ^3^ Anhui Shengjin Lake Wetland Ecology National Long-term Scientific Research Base, Dongzhi, China; ^4^ University of Chinese Academy of Sciences, Beijing, China

**Keywords:** propagule bank, vegetative reproduction, freshwater wetlands, emergent macrophyte, flooding disturbance

## Abstract

Both seed and bud banks play important roles in the recruitment and maintenance of macrophyte communities; however, few studies have investigated them simultaneously. We investigated the abundance, species composition, and seasonal patterns of seed and bud banks in two dominant macrophyte communities, *Carex* and *Miscanthus*, in the Dongting Lake wetlands. The seed densities of both communities were lower from November (after flooding) to March and increased dramatically before flooding (in May). The bud densities of the two dominant communities peaked in the coldest month of the year (January), decreased markedly in March, and were the lowest before flooding. The seed banks of the two macrophyte communities were mainly composed of annual species and a few perennial species, whereas the bud banks were composed of only dominant perennials. Furthermore, the perennial species present in bud banks did not occur in seed banks. Among the soil variables, the bud densities of both plant communities were negatively associated with soil bulk density, whereas the seed density of the *Miscanthus* community was positively associated with soil bulk density. Our results suggest that seed and bud banks are complementary in the potential recruitment of macrophyte communities; that is, bud banks regulate the demography of dominant perennials, and seed banks contribute to the recruitment and dispersal of annual species. Given the high abundance of annuals and near absence of the most dominant perennials in the seed bank, the bud banks of dominant perennial species should be more widely used in wetland restoration and management.

## 1 Introduction

Propagule banks are potential plant communities that include viable seeds in the soil (seed banks) and buds that can potentially be used for vegetative regeneration (bud banks) (Abernethy & Willby, 1999; Chen et al., 2018). The contribution of seed banks to the species composition, recruitment, and successional trends of aboveground communities has long been recognized (Salter, 1856; Kjellsson, 1985; Lokker et al., 1997). However, the importance of bud banks in the regeneration and maintenance of local plant communities has been realized only recently (Harper, 1977; Klimesova and Klimes, 2007; Ott et al., 2019).

In freshwater wetlands and many other herbaceous communities, the seeds of numerous perennial species do not occur in seed banks because they lack viability, undergo dormancy, or experience unsuitable conditions for germination (Sosnová et al., 2010). Therefore, the similarity between seed bank composition and the floristic composition of standing vegetation is relatively low (Hopfensperger, 2007; Cui et al., 2016). The recruitment of these perennial species may primarily depend on vegetative reproduction and bud banks, which most often form on rhizomes, corms, tubers, and bulbs (Klimesova and Klimes, 2007; Dalgleish & Hartnett, 2009; Herben et al., 2016). Although the relative contribution of seed banks versus bud banks to recruitment varies among communities, both seed and bud banks play important roles in the dynamics and structure of macrophyte communities.

Floodplain wetlands are characterized by large fluctuations in water levels and experience completely inundated and exposed periods. Annual species and some perennials may complete their life cycle and produce mature seeds (seed rain) prior to the flooding season (Satake et al., 2001). After flooding, numerous seeds in the soil germinate to replenish extant vegetation (Baskin et al., 2019). Perennial macrophytes may maintain a moderate bud bank during flooding to ensure regeneration after flooding (Vesk and Westoby, 2004; Chen et al., 2015b; Chen et al., 2015c). In addition to flooding, low temperatures constrain the aboveground shoots of live macrophytes through the winter in temperate wetlands. A large reserve of propagule banks in winter may facilitate shoot population recruitment in spring (Chen et al., 2015b). Therefore, seasonal patterns of seed and bud banks play crucial roles in the persistence and dynamics of macrophyte communities.

In freshwater wetlands, zonal distribution of macrophyte communities along elevational gradients is commonly observed (Riis & Hawes, 2002; Chen et al., 2015a). Macrophyte communities distributed at low-elevation sites experience longer inundation duration and more intense flooding disturbance than those at high-elevation sites (Chen et al., 2019). Consequently, a low-elevation distributed macrophyte community may require a larger propagule bank to replace shoots after longer periods of submergence. Because seeds are mobile and can be transported over long distances by water, the seasonal patterns of soil seed banks in floodplain wetlands are diverse (Schneider et al., 2020; Fryirs & Carthey, 2022). As buds are closely associated with parental plants, the seasonal patterns of bud banks along elevational gradients may reflect the adaptation of macrophytes to flooding disturbance (Klimesova and Klimes, 2007; Chen et al., 2015b). However, the seasonal patterns and relative importance of seed and bud banks in propagule banks along elevational gradients are not clear owing to few empirical studies.

In addition to macrohabitat characteristics, the density and composition of seed and bud banks are also influenced by microhabitat characteristics such as soil moisture and fertility (Deng et al., 2013; Chen et al., 2015c). The presence, dispersal, and longevity of buds in a bud bank are closely correlated with the bud-bearing organs of parental plants; therefore, bud banks may be closely related to the habitat factors that affect the growth and development of parent plants (Klimesova and Klimes, 2007). Because the seeds in a seed bank are relatively movable and may persist in the soil even after the vegetation disappears from the site, the seed bank may be less affected by habitat conditions than the bud bank (Baskin et al., 2019). The influence of microhabitat characteristics on propagule bank traits have rarely been quantified in floodplain wetland.

In the present study, we investigated the abundance, species composition, and seasonal patterns of the seed and bud banks in two dominant macrophyte communities in the Dongting Lake wetlands. The wetlands regularly experience a flooding season (June–October) and a cold winter (December–February). In these wetlands, *Miscanthus sacchariflorus* (Maxim.) Hack. (Poaceae) is distributed at higher elevations, and *Carex brevicuspis* C.B. Clarke (Cyperaceae) is dominant in lower elevations (Xie et al., 2015). Specifically, we tested three hypotheses. First, we hypothesized that seed and bud bank densities would be relatively high in the winter because large quantities of seeds and buds are required for aboveground community recruitment in the spring. In contrast, the densities of the seed and bud banks in the two macrophyte communities would be relatively low after flood recession because large quantities of seeds and buds would have germinated into shoots. Second, we hypothesized that the low-elevation distributed *Carex* community would have a larger seed and bud bank than that of the high-elevation distributed *Miscanthus* community because the *Carex* community was more frequently disturbed by flooding, and a larger propagule bank may ensure regeneration after flooding. Third, we hypothesized that the bud banks of the two macrophyte communities would be more closely related to microhabitat characteristics than the seed banks because bud banks are more closely associated with the bud-bearing organs of parent plants.

## 2 Materials and methods

### 2.1 Study sites

Dongting Lake (28°30′–30°20′ N, 111°40′–113°10′ E) is the second-largest freshwater lake in China, with an area of 2625 km^2^. It is located on the southern bank of the middle reaches of the Yangtze River. The surrounding wetlands have a northern subtropical monsoonal climate, with a mean annual temperature of 16.8°C and mean annual precipitation of 1382 mm. The floodplain wetlands experience large water level fluctuations (up to 15 m) and are completely inundated from June to October and exposed from November to May.

### 2.2 Plant communities at the study site

In the Dongting Lake wetlands, *C. brevicuspis* and *M. sacchariflorus* are the most abundant species in the two dominant plant communities, the *Carex* community and *Miscanthus* community, respectively (Xie et al., 2015). *Carex brevicuspis* is distributed at low elevations, ranging from 22–27 m. The pseudoculms of *C. brevicuspis* plants have overlapping leaf sheaths and are usually 20–55 cm in height (Chen et al., 2014). The shoots of *C. brevicuspis* decompose during the flooding season, and new shoots emerge immediately after the flood has receded. In contrast, *M. sacchariflorus* is mostly distributed at elevations greater than 27 m (Xie et al., 2015; Huang et al., 2022). *Miscanthus sacchariflorus* plants are 100–500 cm tall with robust and erect culms (Chen et al., 2015b). The shoots of *M. sacchariflorus* can go through the flooding season and subsequently flower and fruit.

### 2.3 Aboveground and belowground sampling

**Figure 1:**
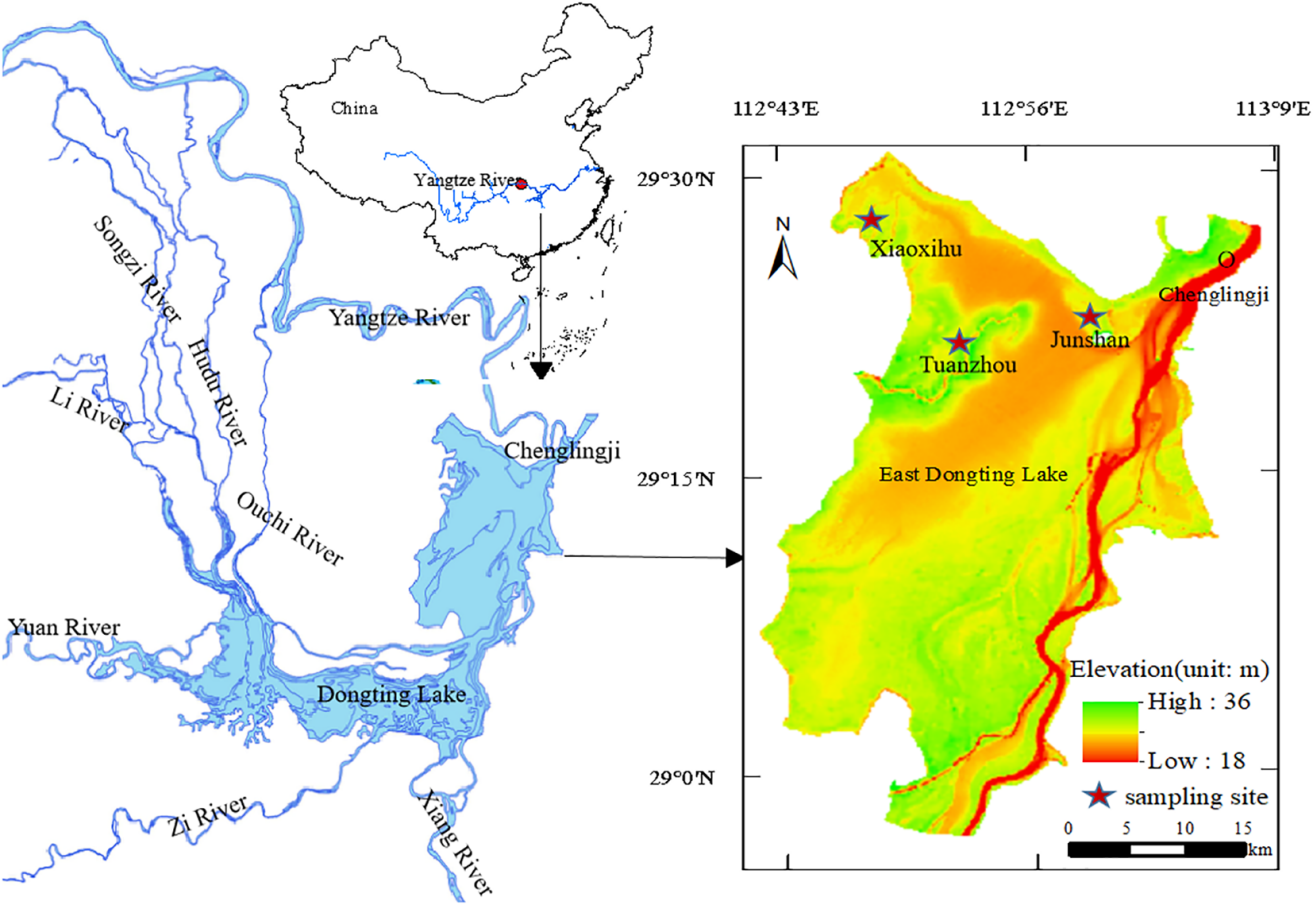
Study area and sampling site locations at East Dongting Lake, China.

Three lake shores where the two communities are typically distributed, Xiaoxihu (112.993684 N, 29.372535 E), Tuanzhou (112.858114 N, 29.336779 E), and Junshan (112.796314 N, 29.458161 E), as study sites (**Figure 1**). A 1 km transect parallel to the lakeshore at each site in the middle of each community zone was established. In November 2017, five permanent plots (5 × 5 m) 100 m apart on each transect at each site was established for sampling aboveground vegetation, soil seed banks, and belowground bud banks. Thirty permanent plots (5 plots × 3 sites × 2 community types) was established. We collected samples after floodwaters receded (in early November 2017), during the coldest month (mid-January 2018), after spring sprouting (early March), and prior to flooding (early May 2018).

#### 2.3.1 Soil seed bank sampling and processing

On each sampling occasion, one quadrat (100 × 100 cm) for soil seed bank sampling within each plot was used. 20 cylindrical soil cores (3.5 cm diameter) from within each quadrat using the auger boring method was took. The soil cores into three sections was divided: upper layer (0–5 cm deep), middle layer (5–10 cm deep), and lower layer (10–20 cm deep). We mixed the 20 cores taken from each depth for each quadrat and combined them into a single soil sample. Overall, there were 45 samples (3 sites × 5 plots × 1 sample × 3 depths) per community type during each sampling period. The seed bank samples were stored at 4°C in a cold closet for at least one month prior to the germination trial.

The abundance and composition of seed banks using the seedling emergence method was assessed (Combroux & Bornette, 2004; Hou et al., 2012). The plastic trays (25 × 20 × 5 cm) was used as germination trays in the greenhouse at the Dongting Lake Station for Wetland Ecosystem Research, Chinese Academy of Sciences. We controlled the temperature at 25 ± 2°C during the day and 17 ± 2°C at night and provided light using 400-W SON-T ARGO sodium lamps (Philips Company, UK) at a photon flux density of 600 µmol m^-2^s^-1^ (PAR) with a 14 h photoperiod. We placed a 3 cm layer of sterilized sand at the bottom of each box. We sterilized the sand at 120°C for 24 h prior to the germination experiment. To remove large debris, we placed the soil seed samples in a shade room, air-dried them, and then sieved them through a fine sieve (0.2 mm mesh). We spread each soil sample evenly over the sand layer, and the soil sample layer was 1 cm thick. Each soil sample was replicated thrice. For each sampling period, there were 270 trays (1 sample × 3 replicates × 3 depth × 5 plots × 3 sites × 2 community types) for seed germination. We watered the trays regularly to keep the surface soil moist and observed seedling emergence every 2–3 d. All the identified seedlings was removed from the trays to prevent competition with newly emerging seedlings. For seedlings those difficult to identify, we culture them independently until they flower, and then identify them according to reproductive traits.

#### 2.3.2 Bud bank sampling and processing

On each sampling occasion, another square quadrat (*Carex* community, 25 × 25 cm; *Miscanthus* community, 50 × 50 cm) was selected for destructive shoot and belowground bud sampling within each plot. We counted and clipped all ramets within the sampling frame in each quadrat. Most rhizomes of the macrophyte species are distributed within the top 10 cm layer of the soil in the Dongting Lake wetlands (Chen et al., 2015b; Chen et al., 2015c). To ensure that all the rhizome buds were collected, the soil was excavated within each frame to a depth of 15 cm using a shovel. Each sample was placed in a plastic bag and transported them to the laboratory. Fifteen quadrats per species was excavated for bud bank sampling on each sampling date.

We carefully cleaned soil from the belowground structures using tap water and then selected rhizomes from each quadrat. We examined belowground buds (rhizome meristems) under a microscope and counted only developed meristems that formed a distinct stem tissue bud (Dalgleish & Hartnett, 2006; Chen et al., 2015b).

#### 2.3.3 Survey of aboveground communities

One quadrat (100 × 100 cm) was established within each plot for the aboveground community survey. On each sampling occasion, the species composition, density, and coverage of each quadrat was surveyed. All aboveground shoots were harvested and belowground root systems were excavated before flooding to obtain biomass. The shoots, roots, and rhizomes was dried separately in an oven at 80°C for 48 h before obtaining the dry weight.

#### 2.34 Soil sampling and processing

Soil corers (100 cm^3^) were used to collect undisturbed sediments for measuring soil bulk density (SBD). In the laboratory, each fresh sediment sample was divided into two parts. One section was used for moisture content (MC) analyses and the other for analyzing the other soil variables. The soil total nitrogen (TN) and total carbon (TC) were measured by elemental analyzer (Elementar, Germany). The soil total phosphorus (TP) was determined by sodium hydroxide melting-molybdenum-antimony resistance colorimetric method (Chen et al., 2015a).

### 2.4 Data analysis

The bud/seed bank density was calculated as the number of buds/seeds per m^2^. We analyzed the differences in seed and bud densities among different sampling seasons and between the two community types using linear mixed models, with community type and sampling season included as the main factors and sampling sites as a random factor. We performed multiple comparisons of means using Tukey’s honestly significant difference (HSD) test at a significance level of 0.05. If necessary, the data was transformed by using square root or log10 to reduce variances’ heterogeneity and tested homogeneity using Levene’s test.

The relationship between seed/bud densities and environmental variables was analyzed using the partial least squares (PLS) regression model, which avoids autocorrelation between independent variables (Huang et al., 2021). The variable importance value (VIP) and model coefficient (MC) were calculated for each independent variable. Variables with VIP values greater than or equal to 0.8 are considered important in the PLS model (Guo et al., 2015). A multiple regression model was used on data that did not conform to the PLS model to analyze the relationship between the dependent and independent variables. All statistical analyses was performed by using the program R (version 3.6.1; R Core Team, 2019).

## 3 Results

### 3.1 Seasonal patterns of seed and bud bank densities

**Figure 2:**
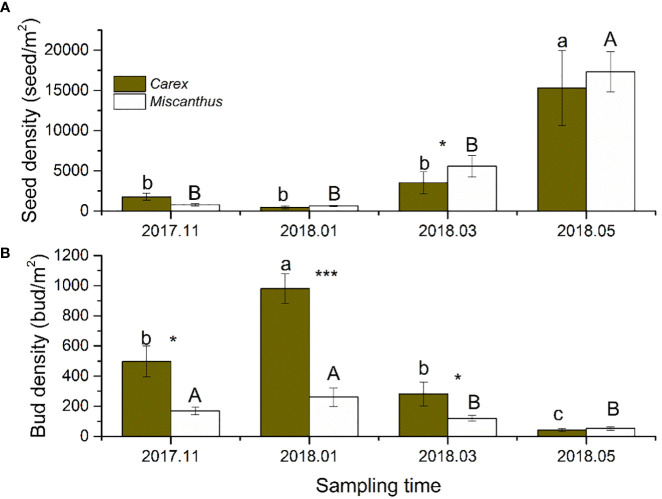
Seed and bud densities of the two dominant macrophytes in four sampling periods. Different lower-case and upper-case letters indicate differences at the 0.05 significance level for *Carex* and *Miscanthus* community respectively. Asterisk indicate significant differences between *Carex* and *Miscanthus* community.^*^ *P* < 0.05, and ^***^ *P* < 0.001.

Seed density varied significantly among sampling seasons, with significant interactions between the sampling season and community type (**Table 1**). The seed densities of the *Carex* and *Miscanthus* communities were relatively low from November (after floodwaters receded) to March (441 ± 165 and 627 ± 98 seeds/m^2^, respectively) and increased dramatically in May (prior to flooding) (15307 ± 4674 and 17330 ± 2486 seeds/m^2^, respectively) (**Figure 2A**). The seed density of the *Carex* community did not differ significantly from that of the *Miscanthus* community, except in March (**Figure 2**).

**Table 1:** Linear mixed model analysis of seed and bud bank densities among different sampling seasons and community types.

Variable	Community (Co)		Season (Se)		Co×Se	
	VE%	*P*	VE%	*P*	VE%	*P*
Seed bank density	0.2	0.650	41.5	0.000	42.2	0.000
Bud bank density	21.0	0.000	29.1	0.000	73.2	0.000
d.f.	1		3		7	

Community type and sampling season were included as fixed factors, and site was included as a random factor. The percentage of variance explained (VE%) and the level of significance (P) are presented.

Bud density varied significantly among sampling seasons and between community types, with significant interactions (**Table 1**). The bud densities of the *Carex* and *Miscanthus* communities peaked in January (980 ± 99 and 261 ± 62 buds/m^2^, respectively), decreased markedly in March, and were the lowest in May (prior to flooding) (41 ± 9 and 52 ± 11 buds/m^2^, respectively) (**Figure 2B**). The bud density of the *Carex* community was significantly higher than that of the *Miscanthus* community in all sampling seasons except May (**Figure 2**).

### 3.2 Species composition of seed and bud banks

In total, there were 23 and 19 species in the seed banks of the *Carex* and *Miscanthus* communities, respectively (**Table 2**). However, the species composition of soil seed banks varied across seasons. In the *Carex* community, the number of species ranged from 6 to 13 and was at its lowest in January. In the *Miscanthus* community, the number of species ranged from 7 to 14 and was the lowest in November.

**Table 2:** Species composition and seed density in the seed banks of *Carex* and *Miscanthus* communities in different sampling periods at the Dongting Lake wetlands.

Species name	Family	Life-form	*Carex* community seed density (seeds/m^2^)				*Miscanthus* community seed density (seeds/m^2^)			
			Nov.	Jan.	Mar.	May	Nov.	Jan.	Mar.	May
*Alopecurus aequalis* Sobol.	Poaceae	annual	396	4	107	1419	38	45	55	239
*Alopecurus aequalis* L.	Apiaceae	annual	76	22	31	164	85	49	523	1060
*Alopecurus aequalis* L.	Brassicaceae	annual	2521	791	6562	25450	1182	452	8785	27948
*Salvia plebeia* R. Brown	Lamiaceae	annual	5	–	121	429	15	78	177	500
*Stellaria* media (Linnaeus) Villars	Caryophyllaceae	annual	9	49	94	1213	28	56	998	732
*Mazus pumilus* (N. L. Burman) Steenis	Scrophulariaceae	annual	517	–	21	784	142	–	1061	1478
*Pseudognaphalium affine* (D. Don) Anderberg	Asteraceae	annual	25	–	–	11	12	–	–	–
*Ranunculus sceleratus* L.	Ranunculaceae	annual	27	–	–	–	–	–	–	–
*Veronica peregrina* L.	Plantaginaceae	annual	43	–	–	–	–	–	–	–
*Hedyotis diffusa* Willd.	Rubiaceae	annual	15	–	–	–	–	–	–	–
*Bothriospermum zeylanicum* (J. Jacquin) Druce	Boraginaceae	annual	23	–	–	–	–	–	–	–
*Phalaris arundinacea* L.	Poaceae	perennial	–	–	3	–	–	26	–	–
*Youngia japonica* (L.) DC.	Asteraceae	annual	–	–	10	–	–	97	104	37
*Leonurus japonicus* Houttuyn	Lamiaceae	annual	–	–	–	–	–	190	31	71
*Galium spurium* L.	Rubiaceae	annual	–	–	42	198	–	7	159	1258
*Paederia foetida* L.	Rubiaceae	perennial	–	–	7	–	–	–	–	–
*Trigonotis peduncularis* (Trev.) Benth. ex Baker et Moore	Boraginaceae	annual	–	–	–	–	–	26	135	202
*Astragalus sinicus* L.	Fabaceae	annual	–	–	–	30	–	67	87	638
*Abutilon theophrasti* Medicus	Malvaceae	annual	–	–	14	–	–	–	59	–
*Hemisteptia lyrata* (Bunge) Fischer & C. A. Meyer	Asteraceae	annual	–	–	–	235	–	–	–	1322
*Clinopodium chinense* (Benth.) O. Ktze.	Lamiaceae	perennial	–	–	–	–	–	41	–	–
*Plantago asiatica* L.	Plantaginaceae	perennial	–	7	–	–	–	246	–	–
*Pharbitis nil* (L.) Choisy	Convolvulaceae	annual	–	11	–	–	–	4	–	–
*Hydrocotyle sibthorpioides* Lam.	Apiaceae	perennial	–	–	–	37	–	–	–	45
*Rumex acetosa* L.	Polygonaceae	perennial	–	–	–	26	–	–	–	–
*Geranium* Rumex acetosa L. *wilfordii* Maxim.	Geraniaceae	perennial	–	–	–	4	–	–	–	–
Species Number			11	6	11	13	7	14	12	13

The soil seed bank of the *Carex* community was composed of 17 annual and 6 perennial species, and that of the *Miscanthus* community was composed of 15 annual and 4 perennial species. The seed density of perennial species was very low, usually less than 50 seeds/m^2^ (**Table 2**). The species present in the bud banks did not occur in the seed banks. Only one species, *C. brevicuspis*, was found in the bud bank of the *Carex* community. There were three species in the bud bank of the *Miscanthus* community: *C. brevicuspis*, *M. sacchariflorus*, and *Phragmites australis*.

### 3.3 Relationships between seed/bud banks and microhabitat characteristics

There were nine annual and seven perennial species in the aboveground *Carex* community, with perennial species accounting for 62.5% of the total shoots (**Table 3**). There were 16 annual and 11 perennial species in the aboveground *Miscanthus* community, with perennial species accounting for 44.8% of the total shoots (**Table 3**).

**Table 3:** Species composition and shoot density in *Miscanthus* and *Carex* communities at the Dongting Lake wetlands.

Species	Family	Life-form	*Carex* community (shoots/m^2^)	*Miscanthus* community (shoots/m^2^)
*Carex brevicuspis* C. B. Clarke	Cyperaceae	Perennial	793	155
*Miscanthus sacchariflorus* (Maximowicz) Hackel	Poaceae	Perennial	0	38
*Phalaris arundinacea* L.	Poaceae	Perennial	9	0
*Lolium perenne* L.	Poaceae	Perennial	3	23
*Alopecurus aequalis* Sobol.	Poaceae	Annual	15	9
*Poa annua* L.	Poaceae	Annual	0	5
*Phragmites australis* (Cav.) Trin. ex Steud.	Poaceae	Perennial	0	9
*Lapsana apogonoides* Maxim.	Asteraceae	Annual	9	22
*Artemisia selengensis* Turcz. ex Bess.	Asteraceae	Perennial	14	25
*Hemisteptia lyrata* (Bunge) Fischer & C. A. Meyer	Asteraceae	Annual	336	11
*Youngia japonica* (L.) DC.	Asteraceae	Annual	0	3
*Stachys japonica* Miq.	Lamiaceae	Perennial	0	6
*Leonurus japonicus* Houttuyn	Lamiaceae	Annual	0	17
*Salvia plebeia* R. Brown	Lamiaceae	Perennial	0	21
*Galium spurium* L.	*Rubiaceae*	Annual	78	114
*Paederia foetida* L.	*Rubiaceae*	Perennial	0	7
*Oenanthe javanica* (Bl.) DC.	Apiaceae	Perennial	2	15
*Daucus carota* L.	Apiaceae	Annual	8	43
*Calystegia hederacea* Wall.	Convolvulaceae	Annual	0	4
*Pharbitis nil* (L.) Choisy	Convolvulaceae	Annual	0	3
*Stellaria media* (Linnaeus) Villars	Caryophyllaceae	Annual	11	54
*Saxifraga stolonifera* Curt.	Saxifragaceae	Perennial	50	31
*Potentilla chinensis* Ser.	Rosaceae	Annual	27	6
*Rumex acetosa* L.	Polygonaceae	Perennial	4	3
*Cardamine hirsuta* L.	Brassicaceae	Annual	31	0
*Trigonotis peduncularis* (Trev.) Benth. ex Baker et Moore	Boraginaceae	Annual	11	46
*Astragalus sinicus* L.	Papilionaceae	Annual	0	58
*Euphorbia helioscopia* L.	Euphorbiaceae	Annual	0	14
*Veronica undulata* Wall.	Scrophulariaceae	Annual	0	2
Total			1401	744

**Figure 3:**
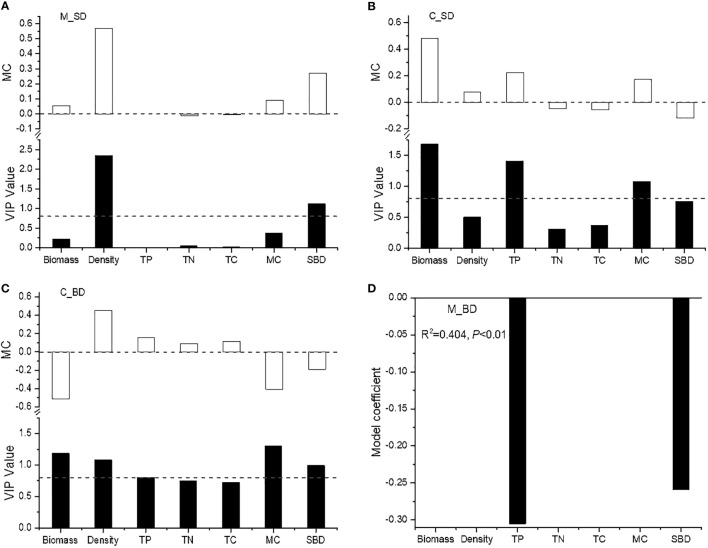
The regression models between seed/bud density and environmental factors. C_SD, seed density of *Carex brevicuspis*; C_BD, bud density of *C. brevicuspis*; M_SD, seed density of *Miscanthus sacchariflorus*; M_BD, bud density of *M. sacchariflorus.* TP, total phosphorus; TN, total nitrogen; TC, total carbon; MC, moisture content; SBD, soil bulk density. **(A)** seed density of *M. sacchariflorus*; **(B)** seed density of *C. brevicuspis*; **(C)** bud doldensity of *C. brevicuspis*; **(D)** bud density of *M. sacchariflorus*.

VIP results showed that the seed density of the *Miscanthus* community was positively associated with aboveground shoot density and soil bulk density (VIP > 0.8; **Table 4**; **Figure 3**). The seed density of the *Carex* community was positively associated with plant biomass, total phosphorus, and moisture content (VIP > 0.8; **Table 4**; **Figure 3**). The bud density of the *Miscanthus* community was negatively correlated with soil total phosphorus and soil bulk density (*P* < 0.05; **Table 4**; **Figure 3**). The bud density of the *Carex* community was positively associated with shoot density and soil total phosphorus, and negatively associated with plant biomass, soil moisture content, and soil bulk density (**Table 4**; **Figure 3**).

**Table 4:** Regression models between seed/bud density and environmental factors.

	Biomass	Density	TP	TN	TC	MC	SBD	R^2^
M_SD	0.054 (0.224)	0.569 (2.353)	-	-0.012 (0.049)	-0.004 (0.019)	0.091 (0.376)	0.272 (1.127)	0.465
M_BD			-0.305^*^				-0.259^*^	0.404^*^
C_SD	0.479 (1.680)	0.079 (0.501)	0.223 (1.404)	-0.048 (0.305)	-0.058 (0.367)	0.171 (1.076)	-0.120 (0.754)	0.342
C_BD	-0.514 (1.188)	0.454 (1.081)	0.159 (0.805)	0.091 (0.746)	0.116 (0.727)	-0.408 (1.302)	-0.191 (0.996)	0.419

Each column represents a different predictor variable (C_SD, seed density of the Carex community; C_BD, bud density of the Carex community; M_SD, seed density of the Miscanthus community; and M_BD, bud density of the Miscanthus community). Significance level: ^*^p < 0.05. a(b) represent model coefficients (MC) and variable importance values (VIP), respectively. (TP, total phosphorus; TN, total nitrogen; TC, total carbon; MC, moisture content; SBD, soil bulk density)

## 4 Discussion

### 4.1 Contrasting seasonal patterns of seed and bud bank densities

Seasonal changes in propagule bank densities and species compositions may indicate when a community will be most resilient or susceptible to disturbance (Ott et al., 2019). The seed densities of both plant communities were relatively constant from November to March and showed a marked increase in May. Variation in the density of soil seed banks in flooded wetlands depends mainly on seed rain, dispersal, germination, and mortality (Nielsen et al., 2018). During the long flooding season, many seeds are lost from the seed bank by flushing, predation, decomposition, and aging (Baskin et al., 2019). Therefore, the seed bank density declined after the flooding in November. Although many seedlings were observed after flooding, such as *Cardamine hirsute*, germination during this period did not cause a significant decline in the seed bank. The seed bank density remained low until May, when abundant seeds, such as *Alopecurus aequalis* and *C. hirsute*, were deposited onto the seed bank.

The bud densities of the two macrophyte communities peaked in winter and declined dramatically after spring sprouting. During the growing season before flooding (March–May), the buds continually sprouted into aboveground shoots, and we observed new ramets of *M. sacchariflorus*, even under the closed canopy. The bud formation rate of parent plants was lower than the rate of bud sprouting; therefore, bud density further decreased before the flooding season. A small bud bank can reduce maintenance costs during a long flooding season (Vesk & Westoby, 2004; Chen et al., 2015b), and it can ensure the regeneration of the aboveground shoots of *M. sacchariflorus* after flooding (Chen et al., 2015d).

Our first hypothesis, which stated that seed and bud bank densities peaked in the winter and were the lowest after the flood recession, was rejected. In the Dongting Lake wetlands, seasonal patterns of seed and bud densities showed nearly contrasting patterns; that is, seed bank density peaked before flooding and was lower in other seasons, whereas bud bank density peaked in the winter and was the lowest before flooding. The contrasting seasonal patterns of seed and bud densities may represent a life-history strategy of wetland plants for adapting to seasonal changes in wetland habitats, such as flooding and cold temperatures (Bao et al., 2018).

### 4.2 The density and species composition of two macrophyte communities

Our results indicate that seed bank density did not differ between the low-elevation *Carex* community and the high-elevation *Miscanthus* community. Seed banks in wetlands are linked to vegetation type, hydrological regime, or other types of disturbances (Abernethy & Willby, 1999; Hou et al., 2012; Greulich et al., 2019). *Carex brevicuspis* flowers, produces seeds and completes the first growing season before the flooding, whereas *M. sacchariflorus* flowers and fruits after the flooding in the fall (Xie & Chen, 2008). Some seeds were directly scattered in litters on the ground, while the others were buried in the soil due to the influence of the external environment (Vesk & Westoby, 2004). In the Dongting Lake wetlands, flood water may flush seeds, sediment, and litter to low-elevation sites when the flood rises (Hou et al., 2012). However, seeds may float for thousands of meters before sinking or being deposited, and deposited seeds may be resuspended and deposited downstream or elsewhere in frequently flood-disturbed floodplains (Gurnell et al., 2008; Fryirs & Carthey, 2022). Therefore, seed bank density may not differ among macrophyte communities along an elevational gradient in the Dongting Lake wetlands.

Nonetheless, we found that the low-elevation distributed *Carex* community had a larger bud bank than that of the high-elevation distributed *Miscanthus* community, which was consistent with a previous study (Chen et al., 2015b). A large bud bank may facilitate rapid regeneration of *Carex* after more frequent flooding disturbance at low-elevation sites, whereas *Miscanthus* may benefit from a small bud bank because of its lower production and maintenance costs and the low probability of flooding disturbance at high-elevation sites (Vesk & Westoby, 2004; Chen et al., 2015b). Overall, our second hypothesis, which stated that the low-elevation distributed *Carex* community has a larger seed and bud bank than the high-elevation distributed *Miscanthus* community, was only partially supported.

### 4.3 Complementary species composition of seed and bud banks

The seed banks of the two macrophyte communities were mainly composed of annual species. Some perennial species, such as *Phalaris arundinacea* and *Paederia foetida*, also appeared in the seed bank, but their densities were very low. The bud banks of the two macrophyte communities were composed of only three perennial species: *C. brevicuspis*, *M. sacchariflorus*, and *P. australis*. Unexpectedly, the three perennial species in the bud bank, which were also the most dominant species in the community, were not present in the seed bank. Consistent with a previous study, no seedling recruitment was found in mature populations of *C. brevicuspis* in the Dongting Lake wetlands (Deng et al., 2015). The absence or rarity of dominant perennials in the seed bank, which may be caused by low seed numbers and/or low seed viability and environmental factors such as flood regime (Fraaije et al., 2015; Cui et al., 2016), has also been observed in other wetlands and grasslands (Galatowitsch & van der Valk, 1996; Benson & Hartnett, 2006).

Therefore, our results suggest that seed and bud banks are complementary in species composition for the recruitment of macrophyte communities; that is, the seed bank ensures the persistence of annual species, and the bud bank enables the regeneration of dominant perennials. Vegetative reproduction *via* bud banks and sexual reproduction *via* seeds may constitute two different strategies for wetland plants to colonize flooding-disturbed wetland habitats (Klimešová and Klimeš, 2007; Herben et al., 2016). Bud banks mediate the demography of dominant perennials and maintain local population processes (Dalgleish & Hartnett, 2009), whereas seed banks contribute to the regeneration of annuals after flooding and are involved in regional processes through seed dispersal (Herben et al., 2016).

### 4.4 Microhabitat factors that influence seed and bud banks

On a large scale, bud density is correlated with precipitation along a climatic gradient in the temperate steppe of northern China and the North American grasslands (Dalgleish & Hartnett, 2006; Qian et al., 2017). At the microsite scale, the soil water status is crucial for bud production, with overly wet conditions reducing the capacity of bud production in *Leymus chinensis* (Zhang et al., 2009). Our results indicate that seed and bud densities were influenced by different soil variables and differed between the two communities. However, the bud densities of both the *Carex* and *Miscanthus* communities were negatively correlated with soil bulk density. The seed bank of the *Carex* community was associated with soil moisture content and soil total phosphorus content, whereas that of the *Miscanthus* community was associated with soil bulk density. These results suggest that soil compactness is an important factor influencing propagule banks in flooded wetlands. Low soil bulk density often implies high soil porosity, aerobic rhizosphere conditions, and high soil organic matter content, which may facilitate rhizome growth and bud production (Deng et al., 2013). The complicated relationships between propagule banks and microhabitat factors need further investigation to understand vegetation succession in response to habitat changes in the context of hydrological and climatic changes.

### 4.5 Implications for wetland restoration and management

To the best of our knowledge, the present study is the first to simultaneously investigate the abundance, species composition, and seasonal patterns of seed and bud banks. We obtained some unexpected results. Seasonal seed and bud densities showed nearly contrasting patterns for the *Carex* and *Miscanthus* communities in the Dongting Lake wetlands. Furthermore, the species composition of the seed and bud banks were complementary. Therefore, both seed and bud banks play important roles in the community recruitment of wetland macrophytes (i.e., bud banks regulate the demography of dominant perennials, and seed banks contribute to the recruitment and dispersal of annual species). The regeneration of macrophyte communities after disturbances depends primarily on the abundance and species composition of propagule banks. In recent years, seed banks have been widely used for wetland restoration (Nishihiro et al., 2006; Hanson et al., 2017). Given the high abundance of annuals and the near absence of most dominant perennials in the soil seed bank, bud banks of dominant perennial species should be introduced during the restoration of wetland vegetation.

## Data availability statement

The raw data supporting the conclusions of this article will be made available by the authors, without undue reservation.

## Author contributions

X-SC: Conceptualization, Methodology, Writing-review and editing. YH: Conceptualization, Methodology, Writing. Z-MD: Conceptualization, Writing-review and editing. FL: Investigation. Z-YH, Y-HC: field survey, data collection. Y-AZ, Y-HX: Methodology. All authors contributed to the article and approved the submitted version.

## Funding

This work was supported by the National Natural Science Foundation of China (31770471, 32071576), the Joint Fund for Regional Innovation and Development of NSFC (U22A20563), Key Research and Development Program of Anhui Province (202104i07020005) the Youth Innovation Development Program of Changsha (kq2106091), Hunan innovative province construction projection (Hunan Key Research and Development Project, 2020NK2012), Youth Promotion Association of Chinese Academy of Sciences (2021365) and Changsha Natural Science Funds for Distinguished Young Scholar (2020).

## Conflict of interest

The authors declare that the research was conducted in the absence of any commercial or financial relationships that could be construed as a potential conflict of interest.

## Publisher’s note

All claims expressed in this article are solely those of the authors and do not necessarily represent those of their affiliated organizations, or those of the publisher, the editors and the reviewers. Any product that may be evaluated in this article, or claim that may be made by its manufacturer, is not guaranteed or endorsed by the publisher.
